# Real-time determination of intracellular oxygen in bacteria using a genetically encoded FRET-based biosensor

**DOI:** 10.1186/1741-7007-10-28

**Published:** 2012-03-22

**Authors:** Janko Potzkei, Martin Kunze, Thomas Drepper, Thomas Gensch, Karl-Erich Jaeger, Jochen Büchs

**Affiliations:** 1 Institute of Molecular Enzyme Technology, Heinrich-Heine-University Duesseldorf, Juelich Research Center, Wilhelm-Johnen-Straße, D-52425 Juelich, Germany; 2 AVT Biochemical Engineering, RWTH Aachen University, Worringerweg 1, D-52074 Aachen, Germany; 3 Institute of Complex Systems-Cellular Biophysics (ICS-4), Juelich Research Center, Wilhelm-Johnen-Straße, D-52425 Juelich, Germany

## Abstract

**Background:**

Molecular oxygen (O_2_) is one of the key metabolites of all obligate and facultative aerobic pro- and eukaryotes. It plays a fundamental role in energy homeostasis whereas oxygen deprivation, in turn, broadly affects various physiological and pathophysiological processes. Therefore, real-time monitoring of cellular oxygen levels is basically a prerequisite for the analysis of hypoxia-induced processes in living cells and tissues.

**Results:**

We developed a genetically encoded Förster resonance energy transfer (FRET)-based biosensor allowing the observation of changing molecular oxygen concentrations inside living cells. This biosensor named FluBO (fluorescent protein-based biosensor for oxygen) consists of the yellow fluorescent protein (YFP) that is sensitive towards oxygen depletion and the hypoxia-tolerant flavin-binding fluorescent protein (FbFP). Since O_2 _is essential for the formation of the YFP chromophore, efficient FRET from the FbFP donor domain to the YFP acceptor domain only occurs in the presence but not in the absence of oxygen. The oxygen biosensor was used for continuous real-time monitoring of temporal changes of O_2 _levels in the cytoplasm of *Escherichia coli *cells during batch cultivation.

**Conclusions:**

FluBO represents a unique FRET-based oxygen biosensor which allows the non-invasive ratiometric readout of cellular oxygen. Thus, FluBO can serve as a novel and powerful probe for investigating the occurrence of hypoxia and its effects on a variety of (patho)physiological processes in living cells.

## Background

Non-invasive detection of intracellular O_2 _is of particular importance since it is one of the key metabolites of obligate and facultative aerobic organisms. Cellular O_2 _is a prominent indicator for oxygen-dependent metabolic activities, such as aerobic respiration or oxygen dependent synthesis and degradation of cellular components [1,2]. In addition, various biological, pathological and biotechnological processes are controlled by O_2 _limitation, including biofilm formation and host-pathogen interactions [3-7], hypoxia induced inflammatory processes [8], tumor pathophysiology [9-12] as well as microbial fermentation processes used for bioremediation and the production of food, feed and biofuels [13-16].

To date, different minimally invasive fluorescence and phosphorescence based O_2_-sensitive probes have been developed for imaging molecular oxygen in cells and tissues. Among them, platinum(II)-porphyrin dyes are widely used for analyzing hypoxia-induced responses of mammalian cells [17-19]. Alternatively, the green fluorescent protein (GFP) and its variants can be applied as genetically encoded intracellular probes that are specifically expressed and can be selectively targeted within defined cells and tissues. In this context, at least two 'passive' GFP-based oxygen sensors have been developed for estimating intracellular oxygen levels in *E. coli*. Here, GFP was applied as a reporter protein expressed under control of oxygen-responsive *E. coli *promoters [20,21]. Additionally, oxygen sensitive photoactivation of GFP-mediated red fluorescence was applied for *in vivo *imaging of oxygen in mammalian cells and organs [22-24].

Remarkably, molecular oxygen can currently not be analyzed *in vivo *by genetically encoded FRET-based biosensors, although these biosensors constitute one of the most widespread classes of fluorescent molecular probes used for the non-invasive quantitative analysis of intracellular compounds including Ca^2+^, Zn^2+^, Cl^-^, pH, H_2_O_2_, ATP, maltose, sucrose, ribose and glucose [25-27]. With respect to oxygen sensing, however, GFP-like proteins, which are commonly used as donor and acceptor domains of FRET biosensors, exhibit a major drawback: Their autocatalytic chromophore synthesis strictly depends on the presence of molecular oxygen [28,29] and thus the fluorescence signal intensity basically does not reflect the amount of synthesized reporter protein [30]. Therefore, GFP and its color variants can not solely be used as fluorescent biosensor domains for accurate O_2 _determination.

Recently, we developed a novel class of fluorescent proteins which carry flavin mononucleotide (FMN) as chromophore [31]. In contrast to GFP-like FPs, the fluorescence signal of these FMN-based fluorescent proteins (FbFP) is independent of cellular oxygen and thus FbFP can be used as quantitative *in vivo *real-time reporter protein under aerobic as well as anaerobic conditions [30,31]. Here, we report the construction and application of the first genetically encoded FRET-based biosensor for oxygen named FluBO which consists of the oxygen-insensitive FbFP donor domain and the hypoxia-sensitive enhanced yellow fluorescent protein (YFP) acceptor domain. We further show that its FRET efficiency dynamically responds to changing O_2 _values in living bacterial cells.

## Results

### Construction and *in vitro *characterization of FluBO

**Figure 1:**
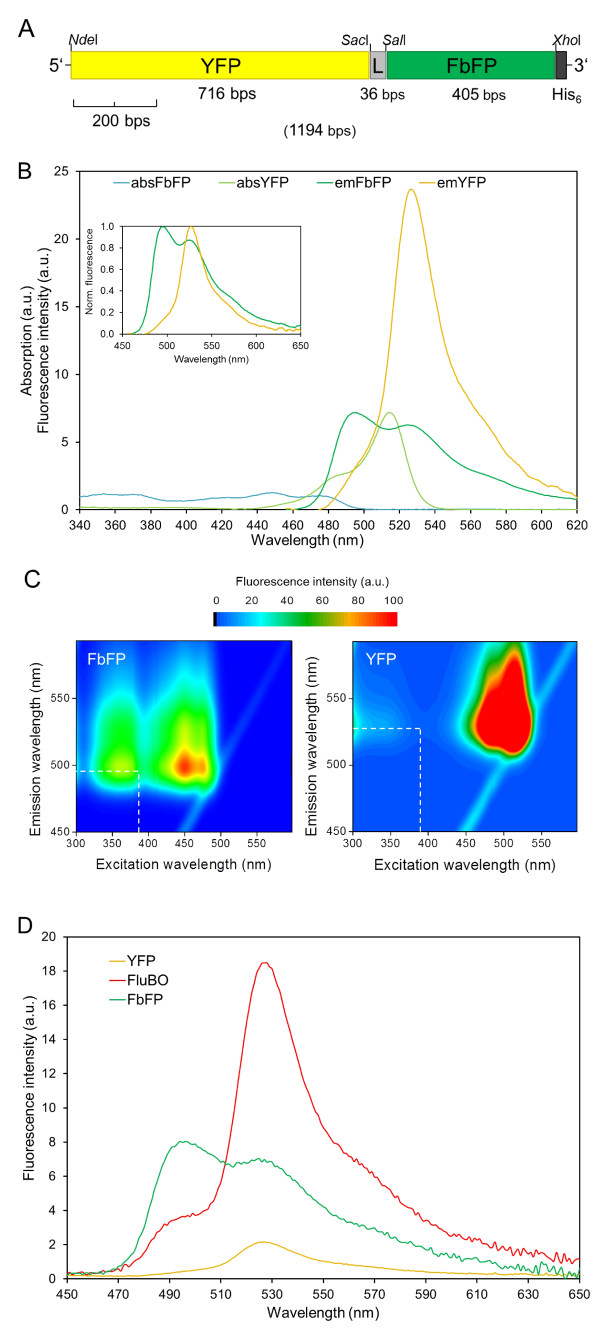
**Design and spectral properties of the FRET-based oxygen biosensor FluBO**. **(A) **Genetic construction of the 1194-bp biosensor gene. The YFP encoding DNA fragment (5'-end) was fused to the FbFP encoding fragment (3'-end) by a 36-bp oligonucleotide which codes for a peptide linker (L) containing a thrombin cleavage site. The restriction endonuclease cleavage sites used for the construction of the biosensor gene are shown. **(B) **Absorption and fluorescence spectra (λ_exc _= 440 nm) of FbFP and YFP in phosphate buffer (pH 8.0, 10 mM NaCl). Absorption spectra are given considering the ratio of the absorption maxima of YFP (514 nm) and FbFP (450 nm) that corresponds to the ratio of the respective extinction coefficients (YFP [35]: 72,000 cm^-1^M^-1^, FbFP: 12,500 cm^-1^M^-1^). Accordingly, the fluorescence spectra were adjusted considering the ratio of the respective fluorescence quantum yields (YFP [35]: 0.76, FbFP: 0.39). The inset additionally shows the corresponding normalized FbFP and YFP fluorescence spectra. **(C) **Two-dimensional wavelength scans of FbFP and YFP. Purified proteins were adjusted to an absorption of 0.1 (λ_FbFP _= 450 nm; λ_YFP _= 514 nm) and fluorescence emission spectra (450 nm to 595 nm) were recorded upon increasing excitation wavelengths from 300 nm to 600 nm. The optimal excitation wavelength, where FbFP but not YFP showed bright fluorescence, is marked by dashed lines. **(D) **Emission spectrum of purified FluBO in comparison to the spectra of donor and acceptor fluorescent proteins FbFP and YFP at λ_exc _= 380 nm; a.u.: arbitrary units. The three proteins were used in equimolar concentrations (approximately 1.5 μM) as judged by the overlap of their absorption spectra in the region > 500 nm (YFP and FluBO) and 320 to 380 nm (FbFP and FluBO).

The *in vivo *fluorescence of YFP, in contrast to FbFP, strictly depends on intracellular oxygen [30]. This observation prompted us to test if a YFP-FbFP fusion can be used as a ratiometric FRET-based biosensor for self-referenced determination of molecular oxygen levels in living cells. The canonical intramolecular FRET-based biosensors are generally fusion proteins consisting of two fluorescent domains with different chromophores that show spectral overlap between the absorption wavelengths of the acceptor domain and the emission wavelengths of the donor domain [32,33]. Therefore, we constructed a recombinant gene (Figure 1A, Additional file 1) encoding the oxygen biosensor which consists of the N-terminal YFP acceptor domain and the C-terminal FbFP donor domain interconnected by a small peptide linker with a thrombin protease cleavage site. Since the enhanced cyan FP (CFP) has most widely been used together with YFP in a tandem FRET biosensor design [32,34], we initially tested whether CFP can be functionally substituted by FbFP as the FRET donor domain. For that purpose, YFP and FbFP as well as the YFP-FbFP fusion protein were separately expressed, purified and their spectral characteristics were analyzed. Figure 1B shows the absorption and emission spectra of FbFP and YFP, respectively. FbFP exhibited its typical absorption spectrum ranging from near UV to blue light with λ_max _at 450 nm, which is characteristic for the FMN chromophore [31]. As required for suitable FRET pairs, the fluorescence emission spectrum of FbFP (λ_max _= 495 nm) substantially overlaps with the YFP absorption spectrum (λ_max _= 512 nm) whereas the emission peaks of FbFP and YFP are sufficiently separated (YFP λ_max _= 528 nm). To minimize direct excitation of the acceptor at the donor excitation wavelength, two-dimensional wavelength scans of FbFP and YFP have been carried out. As demonstrated in Figure 1C, an excitation wavelength of 380 nm results in a bright FbFP fluorescence and a very low excitation of YFP fluorescence. Beside its spectral characteristics, the quantum yield (QY) of FbFP [31] (QY = 0.39) resembles that of CFP [35] (QY = 0.36) indicating that FbFP may be suitable as a FRET donor for YFP.

FRET is a phenomenon of nonradiative energy transfer between the chromophores of the donor and acceptor domain, which can be observed either by a change of (i) ratio of fluorescence intensities emitted by each domain or (ii) lifetime of donor fluorescence. Efficient intramolecular FRET thus results in a decrease of donor fluorescence intensity and of excited-state lifetime. To demonstrate that the YFP-FbFP fusion protein forms a functional FRET pair we first analyzed its fluorescence properties *in vitro*. Fluorescence emission spectra (Figure 1D) of FbFP, YFP and FluBO, respectively, recorded with an excitation wavelength of 380 nm clearly demonstrate that the FbFP fluorescence (green) is largely decreased by efficient energy transfer leading to a dominant fluorescence of the YFP acceptor domain within the fusion protein (red). In contrast, fluorescence intensity of the plain acceptor was low at the same excitation wavelength (yellow). Protease cleavage of the two fluorescent domains resulted in distances much larger than the Förster radius between the donor and acceptor domain and thus in the loss of FRET coupling, which is reflected by a dequenching of the donor FbFP and a dramatic decrease of YFP fluorescence (see Additional file 2).

### *In vivo *functionality of FluBO

**Figure 2:**
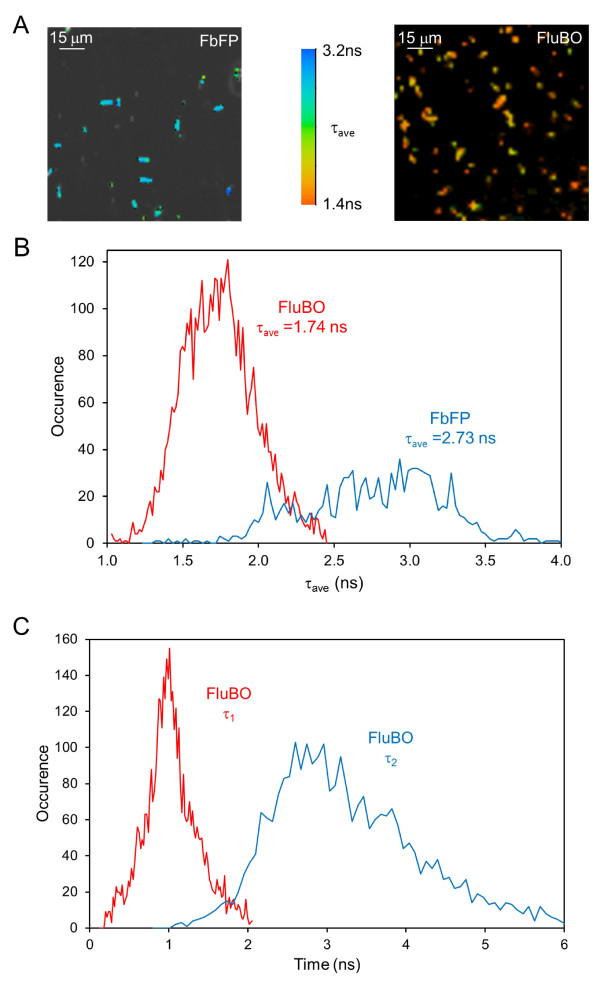
***In vivo *analysis of FluBO fluorescence lifetime**. **(A) **FLIM pictures of *E. coli *cells expressing either FbFP or FluBO. Cells were cultivated until they reached the stationary growth phase. Fluorescence was observed at the maximum of FbFP (λ_obs _< 500 nm; λ_exc _= 760 nm). Two-photon excitation of FluBO fluorescence was tested at different wavelengths and was found best at 760 nm, corresponding the doubled wavelength of the normal one-photon excitation optimum (380 nm, see Figure 1D). The figure shows the fluorescence lifetimes (τ_ave_) of FbFP (left panel) and FluBO (right panel) in living *E. coli *cells. **(B, C) **Analysis of the fluorescence decays of FluBO and FbFP expressed in *E. coli *under aerobic conditions (data are derived from images shown in Figure 2A). The fluorescence decay of FbFP was satisfactorily analyzed with a monoexponential decay function (F(t) = a·exp(-τ_1_/t); τ_ave _= τ = 2.73 ns) while the fluorescence decay of FluBO needed a biexponential decay for correct description (F(t) = a_1_·exp(-τ_1_/t)+ a_2_·exp(-τ_2_/t); τ_ave _= (a_1_·τ_1_+a_2_·τ_2_)/(a_1_+a_2_) = 1.74 ns); τ_1 _= 1.03 ns, τ_2 _= 2.72 ns. Due to FRET, the average fluorescence lifetime τ_ave _of FbFP in FluBO is reduced by 0.99 ns compared to FbFP alone. Using the average lifetimes of FbFP and FluBO and the equation E = 1-τ_ave, FluBO_/τ_ave, FbFP _an apparent FRET efficiency of 37% is calculated (B). The shorter lifetime (mean τ_1 _= 1.03 ns) determined form detailed analysis of the FluBO-FLIM experiments exhibited a major amplitude of 67%. Hence, a FRET efficiency of approximately 62% within the FluBO proteins that undergo FRET can be deduced from τ_1 _(E = 1-τ_1_/τ_ave, FbFP_). The remaining 33% of FluBO molecules behave like single isolated FbFP molecules with a long lifetime of 2.72 ns (C).

Next, FluBO was tested as a FRET-based ratiometric oxygen probe in living cells. Initially, we analyzed the FbFP emission of FluBO in *E. coli *by fluorescence lifetime imaging (FLIM) under aerobic conditions leading to complete maturation of the YFP (that is, the FluBO acceptor domain) chromophore. Figure 2A shows *E. coli *cells expressing either FbFP or FluBO color-coded according to their average fluorescence lifetimes observed at the FbFP fluorescence maximum. Reduction of *in vivo *fluorescence lifetimes of plain FbFP (monoexponential analysis, τ_ave _= 2.73 ns) in comparison to FbFP fused to the FRET acceptor domain YFP (biexponential analysis, τ_ave _= 1.74 ns) (Figure 2A, B) again revealed efficient energy transfer between the donor and the acceptor domain of FluBO with a difference in fluorescence lifetime (Δτ) of 0.99 ns under aerobic conditions. The apparent FRET efficiency (*E*) is 37% using τ_ave _(Figure 2B). A detailed analysis of the FluBO-FLIM experiments in aerated *E. coli *cells revealed that two exponential functions are necessary to describe the observed fluorescence decay behavior satisfactorily (Figure 2C). Here, the longer fluorescence lifetime is very similar to that of FbFP alone. The other, shorter lifetime (mean τ_1 _= 1.03 ns) exhibiting the major amplitude (mean relative amplitude of 67%) represents the FRET process (donor to acceptor energy transfer). Hence, a FRET efficiency of approximately 62% within the FluBO proteins can be deduced from τ_1_. In comparison, the original FRET donor CFP exhibits a similar fluorescence lifetime with a τ_ave _of 2.23 ns whereas FRET-mediated quenching led to an average fluorescence lifetime of 1.67 ns within a YFP-CFP fusion (Δτ = 0.56 ns) and an apparent efficiency *E *of 25.0% [35].

**Figure 3:**
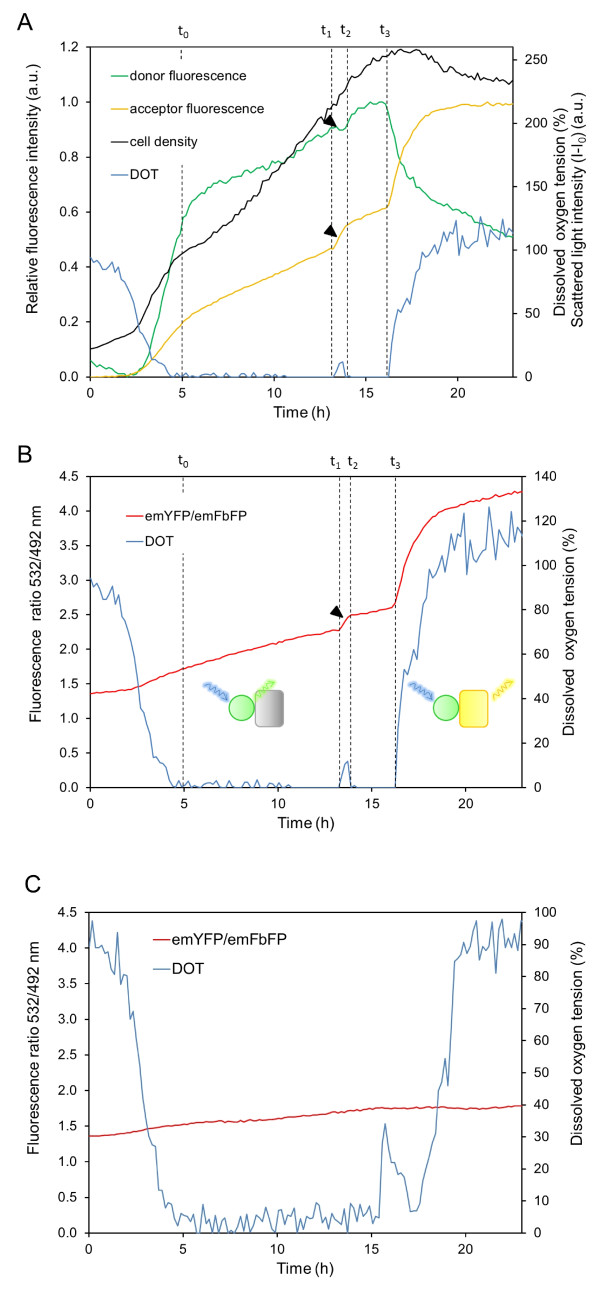
**Determination of intracellular oxygen concentration in *E. coli***. *E. coli *cells expressing FluBO were batch cultured in Overnight Express™ Instant TB medium in 48-well flowerplates using the microcultivation system BioLector. The development of biomass (black), dissolved oxygen tension (blue) and FP-mediated fluorescence (FbFP: green, YFP: yellow) was online-monitored in each well of the flowerplate. Cell density was monitored by scattered light (I-Io) at an excitation wavelength of 620 nm (a.u.: arbitrary units). Time points of changing DOT of the growth medium are labeled (t_0 _to t_3_). Arrowheads indicate changes of donor and acceptor fluorescence which is caused by a temporary shift of DOT during the logarithmic growth phase. **(A) **Fluorescence emission of donor (FbFP) and acceptor (YFP) was recorded at 492 nm and 532 nm, respectively, with an excitation wavelength of 380 nm. **(B) **Development of cyan-to-yellow fluorescent ratio (emYFP/emFbFP) of the FRET-based oxygen biosensor is shown in red. The pictograms symbolize the state of fluorescence activity of the FbFP donor domain (circle) and YFP acceptor domain (rectangle). **(C) **Background fluorescence of *E. coli *during cell growth. *E. coli *cells carrying the empty expression vector were batch cultured in Overnight Express™ Instant TB medium in 48-well flowerplates using the BioLector. Dissolved oxygen tension is shown in blue. According to emFbFP and emYFP, cell-dependent background fluorescence was online recorded at 492 nm and 532 nm that, respectively, at an excitation wavelength of 380 nm and the fluorescence ratio was calculated (red curve). All parallel fermentation experiments in the microwells were performed in triplicates. These results were in excellent agreement (see Additional File 4). Maximum time shifts of 10 minutes occurred due to unavoidable slight differences of cell density at the beginning of the cultivation. Therefore, a representative set of data from these three parallel independent measurements instead of the corresponding mean values is shown in the figures.

Afterwards, we studied the oxygen-dependent fluorescence response of FluBO in *E. coli *cells during batch cultivation (Figure 3A). To this end, the BioLector microbioreactor system (m2p-labs, Aachen, Germany) was used, which provides quantitative online data of the cell density *via *scattered light intensity (black curve) and dissolved oxygen tension (DOT; blue curve) as described previously [30,36,37]. In parallel, intracellular oxygen values of *E. coli *cells expressing FluBO were permanently recorded during cultivation by measuring the donor fluorescence at 492 nm (FbFP, green) and the acceptor fluorescence at 532 nm (YFP, yellow) with an excitation wavelength of 380 nm. After approximately three hours of cultivation, FluBO expression was autoinduced as indicated by rapidly increasing fluorescence intensities of both FP domains. After five hours (t_o_), high respiratory activity of exponentially growing bacteria caused a drastic limitation of O_2 _in the growth medium (DOT ≤ 5%). During this phase of growth, concomitant intracellular oxygen depletion was reflected by a weak increase of YFP fluorescence due to permanently increasing cell numbers and accumulated biosensor protein at low O_2 _concentrations. After 13 hours of cell cultivation, the change of carbon source from glycerol to the overflow metabolite acetate, produced in the first phase of the fermentation, led to a reduced oxygen consumption of *E. coli *cells. Thus, the diauxic growth temporarily elevated the intracellular oxygen value (Figure 3A, t_1_-t_2_) which could be monitored instantly by an increasing YFP fluorescence accompanied by FRET-based decrease of FbFP fluorescence. Consequently, reduced cell respiration led to a transient shift of DOT to a maximal level of 11%. Finally, the DOT level rapidly increased after 16.0 hours when the culture entered the stationary growth phase (t_3_). As expected, this final change of the oxygen concentration also caused an immediate increase of YFP fluorescence accompanied by a decrease of FbFP fluorescence.

The YFP-FbFP fusion construct FluBO represents a FRET pair and thus provides a fixed stoichiometry of the two FP domains. Hence, the ratio of observed YFP and FbFP fluorescence emissions directly reflects the change of cellular oxygen concentration (Figure 3B). Remarkably, extra- and intracellular O_2 _values are directly correlated and developed cooperatively during cell cultivation. For instance, low DOT which occurred in the late logarithmic growth phase (Figure 3A, B; t_2_-t_3_) resulted in an almost constant YFP-to-FbFP fluorescence ratio (Figure 3B) until the molecular oxygen concentration increased due to the physiological adaptation of the bacteria during the stationary growth phase. Here, the change of intracellular oxygen availability is directly reflected by an increasing FluBO fluorescence ratio. The ratiometric change of donor and acceptor fluorescence is caused by O_2_-mediated maturation of the YFP chromophore resulting in dominant YFP fluorescence and a FRET-dependent quenching of FbFP fluorescence (Figure 3A, B). However, bacterial proteins exhibiting an intrinsic fluorescence may affect the biosensor-based oxygen readout. Therefore, an *E. coli *strain carrying the corresponding empty expression vector was cultivated under the same conditions and the ratio of cell-mediated background fluorescence was analyzed at identical wavelengths (Figure 3C). Here, an only marginal change of the emission ratio was detected, which was clearly independent of the DOT level.

### *In vivo *calibration of the fluorescent protein-based oxygen biosensor

**Figure 4:**
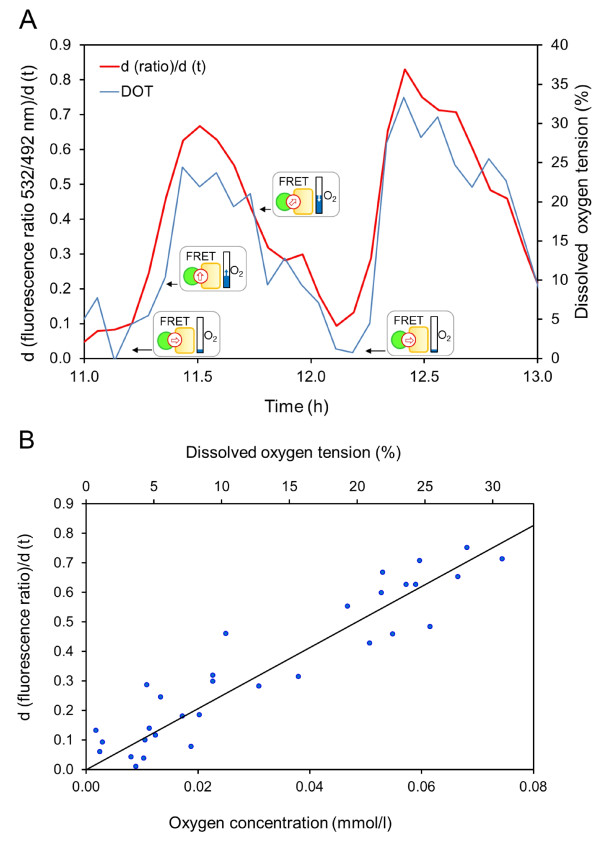
**Calibration of FluBO for quantitative determination of intracellular oxygen**. **(A) **During the logarithmic growth of *E. coli *cells, oxygen limitation was temporarily relieved twice by increasing the shaking frequency. The fitting curve of the time derivation of cyan-to-yellow fluorescence ratio (red) was drawn as described in the Methods. The pictograms symbolize the velocity of change of FRET ratio in dependence on cellular oxygen values: ⇨, no change; ⇧, fast increase; ⇗, slow increase. **(B) **The same data have been used to generate the calibration curve. In the used medium the value of 100% DOT corresponds to an oxygen concentration of 0.24 mmol/l at 30°C and an atmospheric pressure of 100 kPa. All parallel fermentation experiments in the microwells were performed in triplicates. The figures show a representative set of data.

The results presented so far clearly demonstrate that the development of DOT directly correlates with intracellular oxygen levels basically allowing the *in vivo *calibration of the FRET biosensor within the used *E. coli *test system. However, the emission ratio of FluBO at a certain time point of cell growth is modulated by the availability of oxygen as well as by the time needed for FluBO synthesis and YFP chromophore formation. Furthermore, oxygen-dependent maturation of the YFP chromophore is an irreversible process. Therefore, repeating changes from sufficient to deficient oxygen concentrations as occurring during batch cultivation of *E. coli *indeed resulted in the inhibition of YFP chromophore maturation and thus led to almost constant but stepwise increased cyan-to-yellow fluorescence ratios under anaerobic conditions (Figure 3B). For that reason, we subsequently considered the change of FluBO fluorescence ratio over time (d (ratio)/d (t)) as a measure for fluctuating intracellular O_2 _concentration. To accurately calibrate FluBO *in vivo, E. coli *cells were batch cultured to the mid-logarithmic growth phase (that is, to oxygen limited conditions) and, subsequently, DOT was temporarily increased twice. As shown in Figure 4A the change of FluBO emission ratio over time directly corresponded to the respective DOT levels. Remarkably, with the onset of repeated O_2 _deprivation in the medium, lowering of intracellular oxygen level led to a retarded YFP chromophore maturation, which in turn was directly reflected by a decreasing (d (ratio)/d (t)) value of FluBO fluorescence. Hence, this new method also allows the indirect detection of decreasing oxygen levels in living cells. Finally, a linear oxygen calibration curve could be derived from the *E. coli in vivo *fluorescence data (Figure 4B) covering a DOT level from 0 to 33% corresponding to a molecular O_2 _concentration up to 0.08 mmol/l.

## Discussion

### Characteristics of the oxygen biosensor FluBO

Here, we describe for the first time the development of a genetically encoded FRET-based biosensor, allowing the determination of molecular oxygen inside live cells. The biosensor FluBO consists of the cyan fluorescing flavoprotein FbFP and the yellow fluorescing GFP derivative YFP as respective FRET donor and acceptor domains. Here, the oxygen demand for chromophore formation of YFP, but not of FbFP is the basis for the detection of cellular oxygen changes *via *the O_2_-dependent alteration of FRET efficiency. At atmospheric levels of O_2_, YFP chromophore maturation leads to efficient FRET coupling causing an efficient quenching of FbFP fluorescence and a dominant YFP fluorescence. In contrast, the absence of molecular oxygen prevents YFP chromophore formation and thus immature YFP has no effect on the fluorescence of the FbFP donor domain due to the loss of FRET coupling. To evaluate the *in vivo *functionality of FluBO, we extensively analyzed the biosensor in *E. coli *cells during batch cultivation. Parallel online detection of extra- and intracellular oxygen levels using an oxygen sensitive optode and the genetically encoded biosensor revealed a clear correlation between O_2 _levels outside and inside the cells.

### Calibration of FluBO

Development of DOT directly correlates with intracellular O_2 _levels hence allowing the *in vivo *calibration of the FRET biosensor within the used *E. coli *test system. The resulting oxygen calibration curve was derived from the FluBO (d (ratio)/d (t)) values that correspond to defined concentrations of extracellular oxygen.

Generally, novel intramolecular FRET-based biosensors are pre-calibrated *in vitro *and subsequently transferred into the living system of interest [38-41]. However, in contrast to other FP-based biosensors which allow the quantification of a certain metabolite, *in vitro *calibration of FluBO is not an easy task. Accurate pre-calibration requires biosensor protein, which was expressed and subsequently purified under anaerobic conditions. In addition, *in vitro *calibration has to be conducted using the gaseous substrate O_2 _in an otherwise oxygen-free atmosphere. Furthermore, biosensor pre-calibration basically does not take into account that differences in non-specific interactions between a molecule (that is, the respective biosensor) and its immediate surrounding (for example, a buffer in comparison to the interior of a living cell) can greatly influence the equilibrium and rate of the respective reaction [42,43]. For that reason, the use of pre-calibrated reference probes does not inevitably lead to accurate *in vivo *calibration of a novel biosensor. Finally, as discussed below, the level of biosensor expression and, to a minor extent, its stability (or turnover) in the respective host cell rather than the cellular growth rate most probably represents the major bottleneck for functionality of a genetically encoded biosensor. However, these factors significantly depend on the individual properties of a biological system and might even vary within a particular cell depending on growth conditions and/or environmental parameters. Additionally, ratio of extra- and intracellular oxygen concentrations in different organisms might vary in dependence on O_2 _consumption, which in turn can also be affected by individual parameters, such as variable metabolic activities and cell organization as well as heterogeneity of oxygen diffusion within cellular compartments and tissues. Consequently, the intracellular O_2 _concentration inside *E. coli *cells is probably much lower than in their surrounding medium (reflected by DOT). Therefore, the applicability of an intracellular biosensor as a quantitative probe needs to be evaluated and calibrated separately for each organism and condition to achieve reliable data.

### Environmental sensitivity of FluBO

Most of the YFP derivatives described in the literature exhibit a distinct pH sensitivity with pK_a _values ranging from 6.9 (EYFP) to 5.7 (mCitrine) [28]. Using YFP as a FRET receptor domain, therefore, ideally requires a constant fluorescence brightness which is not affected by intracellular changes of pH. To quantitatively interpret oxygen measurements with FluBO, we utilized an EYFP variant distributed by Clontech-Takara (details are described within the Methods section and the corresponding EYFP sequence is shown in Additional file 1). Analysis of pH sensitivity demonstrated that this YFP derivative exhibits a remarkable pH resistance (pK_a _= 5.2; Additional file 3). Thus, FluBO-based oxygen determination is not affected within the physiological pH range. In addition, the early YFP variants were also sensitive towards chloride [28]. To further rule out that chloride ions influenced the FluBO oxygen response *in vivo*, we also determined Cl^- ^sensitivity of the used EYFP. We could demonstrate that YFP fluorescence intensity remained constant in the presence of chloride concentrations up to 100 mM (data not shown). This result clearly indicates that FluBO is also insensitive to changes of chloride-ion concentrations in the physiological relevant range.

### Limitations of FluBO

Although FluBO is a useful molecular probe, some aspects must be considered to allow online-detection of intracellular oxygen: First, as mentioned above, oxygen-dependent chromophore formation is an irreversible process. Consequently, this biosensor indeed allows analyzing changes from low to high oxygen concentrations but it cannot directly be used to detect reduction of oxygen levels *via *its change of intramolecular FRET efficiency. Therefore, we analyzed alterations from sufficient to deficient oxygen levels by observing the total changes of dual emission ratio over time. By using this method, recurring oxygen limitation was reflected by a stagnating alteration of YFP-to-FbFP fluorescence ratio (as for example, visible in Figure 3B between t2 and t3) and in turn by decreasing (d (ratio)/d (t)) values (Figure 4A). Furthermore, it should be emphasized that due to the irreversibility of O_2_-mediated YFP chromophore formation repeated changes or a long-lasting increase of intracellular oxygen level can be determined only under specific conditions. Namely, the degradation of oxidized FluBO and the production rate of non-oxidized FluBO need to form an equilibrium ensuring a continuous and sufficient supply of biosensor molecules which did not 'sense' oxygen before. It, therefore, remains to be experimentally demonstrated how FluBO behaves in cells exhibiting significantly lower metabolic activity than *E. coli*. Nevertheless, we expect the FluBO (d (ratio)/d (t)) value to represent a valid indication of intracellular oxygen levels in a large variety of different (micro)organisms. To this end, we are currently analyzing the applicability of FbFP and the FbFP-based biosensor FluBO in various prokaryotic and eukaryotic including mammalian cell lines.

### Alternative *in vivo *O_2 _sensors

Monitoring the heterogeneity and development of intracellular oxygen levels with high spatio-temporal resolution requires a molecular O_2 _biosensor that exhibits some important properties. For example, the ideal oxygen indicator must be either uniformly distributed or, alternatively, can be specifically targeted within a cell or tissue of interest. For quantitative *in vivo *O_2 _determination, the biosensor has to respond rapidly, reliably and reversibly to the full range of physiological oxygen concentrations.

In the last decade, different fluorescent dyes and proteins have been developed, which can be used as sensors for intracellular oxygen measurements.

(1) Various fluorescence and phosphorescence based O_2_-sensitive probes, including the complex platinum(II)-porphyrin dyes are widely used for imaging molecular oxygen in living organisms [17-19]. These probes exhibit a selective and reversible response to O_2 _within the full physiological oxygen range (0 to 250 μM) combined with optimal photophysical properties (for example, high fluorescence brightness). Nevertheless, in comparison to genetically encoded sensors intracellular loading of these dyes appears to be relatively inefficient, slow and, furthermore, they cannot be generally targeted to specific cells or intracellular compartments [19,44,45]. Consequently, intracellular visualization of O_2 _with high spatio-temporal resolution is limited.

(2) Alternatively, GFP can be utilized as a reporter gene, whose expression is under control of an oxygen sensitive regulator within a certain organism. For example, in *E. coli *promoters P_*nar *_(controlling nitrate reductase expression) and P_*rpoS *_(controlling the expression of the stress response sigma factor σ^S^), which are activated under microaerobic and anaerobic conditions, were applied to monitor oxygen limitations [20,21]. Although this technique is potentially capable of analyzing limited oxygen supply and differences in population heterogeneities, this biosensor architecture can only be applied for specific host organisms. Furthermore, regulators are often part of a higher-order regulatory network and thus might not solely respond to oxygen deprivation. Beside oxygen sensitive GFP reporter gene constructs, Takahashi and coworkers described an exceptional technique that also allows GFP-dependent *in vivo *oxygen determination [22-24]. This approach makes use of the oxygen sensitive red-shifted fluorescence of GFP that occurs after photoactivation with blue light [46]. However, since the magnitude of the observed red shift is abruptly increased with oxygen concentrations < 1%, this technique can only be applied for measuring low intracellular oxygen levels. Furthermore, the basic mechanism of GFP photoactivation of red fluorescence as well as the oxygen-dependent recovery to green fluorescence is not fully understood yet. Finally, it is worth mentioning that both of the described strategies, using GFP either as 'passive' or 'active' oxygen biosensor, inherently suffer from impaired chromophore formation under oxygen limited conditions. This fact makes FluBO the first fluorescent protein-based biosensor enabling exact and reliably determination of molecular oxygen.

## Conclusions

Accurate determination of intracellular oxygen and analysis of changing O_2 _levels inside living cells and tissues is of the utmost importance in gaining new insights into complex (patho)physiological processes associated with oxygen deprivation, including invasion and persistence of pathogens or tumor development. Our results show that FluBO can be used as a ratiometric biosensor for molecular oxygen that changes the emission maxima of the donor and acceptor domain as a function of O_2 _availability and its sensitivity is within the physiological range relevant for bacteria as well as for eukaryotes, including mammalian and human cells [2]. The oxygen modulated FRET-coupling allows the non-invasive readout of cellular O_2 _levels as well as the online analysis of its changes with high spatial and temporal resolution by fluorescence spectrometry and FLIM. Thus, the observation of cellular oxygen levels in any cellular compartment using genetically encoded FRET biosensors will offer a novel rational approach to understand hypoxia-dependent processes in biological, biomedical and biotechnological processes.

## Methods

### Bacteria and growth conditions

*E. coli *strain DH5α [47] was used for DNA cloning of the expression vectors encoding the fluorescent reporter proteins FbFP and YFP, as well as the biosensor FluBO. *E. coli *strain BL21(DE3) (Novagen, distributed by Merck KGaA, Darmstadt, Germany) was used for expression of the fluorescent proteins and FRET-based biosensor FluBO. For protein expression and purification, bacterial cells were grown either in autoinduction TB medium consisting of 5 g/l glycerol, 12 g/l tryptone, 24 g/l yeast extract, 2.32 g/l KH_2_PO_4_, 12.5 g/l K_2_HPO_4 _(pH 7.2), lactose 2 g/l, glucose 0.5 g/l (for FbFP expression and purification) or in LB medium consisting of 10 g/l tryptone, 5 g/l yeast extract, 10 g/l NaCl (for YFP expression and purification). BioLector cultivation experiments were conducted in Overnight Express™ Instant TB medium (Novagen) as described below. All media were supplemented with 50 μg/l kanamycin to maintain expression vector pRhotHi-2.

### Construction of FP expression vectors

The construction of expression vector pRhotHi-2-YFP encoding the enhanced yellow fluorescent protein YFP (available from Clontech-Takara Bio Europe, Saint-en-laye, France) and pRhotHi-2-FbFP encoding EcFbFP (GenBank number: ABN71355; commercially available as evoglow-Bs2, evocatal GmbH, Düsseldorf, Germany) was described previously [31]. The YFP variant used in this study contains four amino acid substitutions previously published as GFP-10C [48], as well as the substitution H231L and the insertion of valine behind the first amino acid. The expression vector pRhotHi-2-FluBO harboring the FRET-based oxygen biosensor encoding gene was constructed using a synthetic FluBO gene (Eurofins MWG Operon, Ebersberg, Germany) whose DNA sequence is shown in Additional file 1. The biosensor gene was cloned into the *Nde*I and *Xho*I restriction sites of pRhotHi-2 [49] and recombinant FP genes were confirmed by DNA sequencing. The pRhotHi-2-FluBO vector will soon be commercially available (evocatal GmbH, Düsseldorf, Germany).

### Protein expression and purification

*E. coli *BL21(DE3) cells carrying the expression plasmids pRhotHi-2-FbFP, pRhotHi-2-YFP and pRhotHi-2-FluBO, respectively, were grown until the cultures reached an optical density of approximately 0.5 at 580 nm. Protein expression was then induced by adding 1 mM isopropyl-β-D-thiogalactopyranoside (IPTG) to the culture medium. Recombinant oxygen biosensor FluBO, YFP and FbFP were purified as His_6_-tagged proteins from *E. coli *BL21(DE3) after 16 h of expression at 37°C. Purification was carried out by using HisTag Superflow Hightrap gravityFlow cartridges (Qiagen, Hilden, Germany) and peristaltic pump P-1 (GE Healthcare Europe GmbH, Munich, Germany) under standard operation conditions as described by the manufacturers. The purified proteins were stored at 4°C in protein storage solution containing 10 mM NaCl, 10 mM NaH_2_PO_4_, pH 8.0.

### Spectral analysis and fluorometry

The absorption and fluorescence properties of FbFP, YFP and FluBO were determined in 10 mM NaCl and 10 mM sodium phosphate buffer pH 8.0 on a UV-2450 absorption spectrophotometer (Shimadzu Europa GmbH, Duisburg, Germany) and a QM-4 fluorescence spectrophotometer (PTI, Ford, West Sussex, UK), the latter with spectral correction for detector and emission monochromator transmission. The absorption spectra were recorded by scanning from 330 nm to 620 nm; the emission spectra were recorded at excitation wavelengths of 380 nm or 440 nm. When emission spectra were measured the maximum absorption of the samples did not exceed 0.15. In the case of excitation/emission (2D) scans, fluorescence excitation was determined from 300 nm to 600 nm in 5 nm steps by using the PerkinElmer LS50B Luminescence Spectrometer (Wellesley, MA, USA) at 22°C. Here, the fluorescence emission spectra were detected from 450 nm to 595 nm wavelength.

### Thrombin protease cleavage

For the thrombin protease mediated cleavage of the FluBO fusion protein, 10 μg of purified protein and one unit of thrombin protease (Novagen, distributed by Merck KGaA, Darmstadt, Germany) were diluted in 50 μl thrombin cleavage buffer. After incubation at room temperature for 16 hours an aliquot of 10 μl was used for SDS-PAGE analysis to corroborate the cleavage of the YFP-FbFP fusion by thrombin protease. SDS-PAGE gels were prepared using standard methods (NuPAGE, 4 to 12% Bis-Tris gel 1 mm, Invitrogen, Paisley, UK). After SDS-PAGE separation, proteins were transferred to a PVDF membrane (Bio-Rad Laboratories GmbH, Munich, Germany) by Western blotting (NuPAGE, Invitrogen, Paisley, UK). Subsequently, YFP and FbFP proteins were detected using the respective antibodies (rabbit anti-GFP, BD Biosciences Erembodegem, Belgium; YtvA-specific antiserum). To detect the YFP-FbFP fusion protein FluBO, a His-tag antibody (Anti-His-HRP antibody, Invitrogen, Paisley, UK) was used. Immunodetected proteins were visualized using a CCD camera-based chemiluminescence detection system (Stella imaging system with AIDA advanced image data analyzer, Raytest, Straubenhardt, Germany). Fluorescence emission of FPs was determined by using the PerkinElmer LS50B Luminescence Spectrometer at 22°C as described above.

### Fluorescence lifetime imaging and analysis

The *E. coli *cells expressing FbFP, YFP or FluBO were placed in suspension between two glass slides on the stage of an upright fluorescence microscope (BX50WI; Olympus Optical, Tokyo, Japan) and observed through a 60 × water-immersion objective (NA = 0.9; Olympus Optical). Fluorescence was excited with ca. 100 fs light pulses (λ_exc _= 760 nm) applied at sufficient intensity to generate two-photon excitation. Light pulses were generated at a frequency of 80 MHz by a mode-locked Titan-Sapphire laser (MaiTai DeepSee; output power > 2W; Newport, Spectra Physics Irvine, CA, USA). The laser light was directed through the lens onto the *E. coli *at reduced power (ca. 5 mW) and scanned over the sample using a beam scanner (TILL Photonics, Munich, Germany). Fluorescence was recorded by a photomultiplier (PMT-100, Becker & Hickl, Berlin, Germany) using appropriate filters for detecting the FbFP fluorescence of FbFP and FluBO (short pass filter 500 nm; λ_obs _< 500 nm; Omega Optical, Brattleboro, VT, USA) and the YFP fluorescence of YFP and FluBO (band pass filter 535 +/- 13 nm; Omega Optical). Fluorescence lifetime imaging was performed using electronics for time-correlated single photon counting (Simple-Tau 152; Becker & Hickl) and acquisition software (SPCM8.95; Becker & Hickl) for time-correlated single-photon counting as described before [50,51]. Lifetime images were analyzed using SPCImage 2.97 (Becker & Hickl) by fitting mono- or biexponential model functions to the fluorescence decays in every pixel of the image. The program uses an iterative reconvolution of the exponential function with an instrument response function and a least square algorithm for finding parameters of the exponential function for a satisfactory fit.

### Characterization of the oxygen biosensor using the BioLector microbioreactor system

All cultivations were carried out in sterile black 48-well microtiter plates (Flowerplate, m2p-labs, Aachen, Germany) in the microcultivation and on-line monitoring system BioLector (m2p-labs, Aachen, Germany) [36,37]. These microtiter plates allow higher oxygen transfer rates as compared to conventional microtiter plates [52]. Furthermore, each well is equipped with an optode for the determination of DOT (% air saturation) *via *fluorescence measurement. The microtiter plates were sealed with sterile gas-permeable adhesive seals (Thermo Scientific, Waltham, MA, USA), ensuring sterile conditions in the wells and allowing good ventilation. The following conditions were applied for all cultivations in the BioLector: temperature 30°C, total filling volume per well: 600 μl, shaking diameter: 3 mm, relative humidity in the incubation chamber: 80%. For all cultivations Overnight Express™ Instant TB medium (Novagen, distributed by Merck KGaA, Darmstadt, Germany) was used. Cultures were inoculated from pre-cultures to an initial OD_600 _of 0.1. Pre-cultures were made in 250 ml flasks under the following conditions: inoculation from a cryoculture to yield an initial OD_600 _of 0.1, temperature 37°C, total filling volume 10 ml of TB medium, shaking diameter 50 mm, shaking frequency 350 rpm, growth overnight to stationary phase. For the batch cultivation in the BioLector the shaking frequency was 800 rpm. For DOT shift experiments the initial shaking frequency was 600 rpm, which was increased to 700 rpm after 11.2 h and 800 rpm after 12.2 h. The biomass (I-Io) was measured via scattered light intensity at an excitation wavelength of 620 nm. Donor (FbFP) and acceptor (YFP) fluorescence were excited at 380 nm and emission was detected at 492 nm and 532 nm, respectively. DOT was measured at an excitation wavelength of 505 nm and an emission wavelength of 590 nm. The measuring cycle was 10 minutes for the batch cultivation and 4.5 minutes for the DOT shift experiment. Parallel fermentation experiments in the microwells were performed in triplicates. These results were in excellent agreement. Maximum time shifts of 10 minutes occurred due to unavoidable slight differences of cell density at the beginning of the cultivation. Therefore, a representative set of data from these three parallel independent measurements is shown instead of the corresponding mean values.

### Data handling

For the calibration of the optode-derived DOT signal and the FRET signal we considered the derivation of the YFP-FbFP ratio over the time (d (ratio)/d (t)). In this way, it is possible to also follow decreasing DOT values. To reduce the influence of measuring errors for the calculation of d (ratio)/d (t) the curves for the FluBO fluorescence ratio were fitted to the raw data. This was done in MATLAB 7.11 (The MathWorks, Inc., Natick, MA, USA) applying a smoothing spline with a smoothing parameter of 0.999. In the used medium the value of 100% DOT corresponds to an oxygen concentration of 0.24 mmol/l at 30°C and an atmospheric pressure of 100 kPa.

## Abbreviations

ATP: adenosine triphosphate; a.u.: arbitrary units; DOT: dissolved oxygen tension; FbFP: flavin-binding fluorescent protein; FLIM: fluorescence lifetime imaging; FluBO: fluorescent protein-based biosensor for oxygen; FMN: flavin mononucleotide; FP: fluorescent protein; FRET: Förster resonance energy transfer; GFP: green fluorescent protein; IPTG: isopropyl-β-D-thiogalactopyranoside; PVDF: polyvinylidene fluoride; QY: quantum yield; SDS-PAGE: sodium dodecyl sulfate polyacrylamide gel electrophoresis; UV: ultraviolet; YFP: yellow fluorescent protein.

## Competing interests

The authors declare competing financial interests. Parts of the results described in this publication have been included into a patent application (Circolone F., Drepper T., Endres S. Heck A., Jaeger K.-E., Potzkei J. (2010); Patent number DE 10 2010 037 001). The oxygen biosensor FluBO will be commercialized by evocatal GmbH, Germany.

## Authors' contributions

TD developed the idea for the project and designed the experiments concerning the construction and characterization of the biosensor. JP designed and performed the experiments concerning the construction and characterization of the biosensor. MK and JB designed and performed the BioLector experiments. TG designed and performed the FLIM experiments. JP, MK, TG, TD and JB developed the method for data processing and analysis. TD and K-EJ wrote the paper. All authors read and approve the final manuscript.

## Supplementary Material

Additional file 1 **FluBO nucleotide and amino acid sequence**. Yellow, YFP sequence; black, linker sequence; green, FbFP sequence; grey, His_6 _tag sequence. Click here for file

Additional file 2 **Fluorescence spectra of FluBO before and after proteolysis with thrombin**. Fluorescence spectra of FluBO before and after proteolysis with thrombin. The fluorescence emission spectra of purified FPs (5 μM) were recorded at an excitation wavelength of 380 nm. Complete thrombin-catalyzed proteolysis of FluBO was demonstrated by SDS-PAGE (inset), purified FPs were used as standards. Click here for file

Additional file 3 **Sensitivity of used YFP towards pH**. The figure shows the normalized YFP fluorescence intensity at increasing pH values. Purified YFP was adjusted to an absorption of 0.05. The effect of the pH value on YFP fluorescence was measured in citric acid/disodium phosphate buffer with pH values ranging form 4.0 to 7.0. The pK_a _value is marked by doted lines. Click here for file

Additional file 4 **Data from parallel fermentation experiments**. The figure shows the individual results from three independent batch cultivation experiments using the BioLector. The development of biomass (black), dissolved oxygen tension (blue) and cyan-to-yellow fluorescent ratio (emYFP/emFbFP) of the FRET-based oxygen biosensor (red) was online-monitored in each well of the flowerplate. Click here for file
