# Supplementary material for: Real-time determination of intracellular oxygen in bacteria using a genetically encoded FRET-based biosensor
Source: BMC Biol. 2012 Mar 22;10:28. doi: 10.1186/1741-7007-10-28 (PMC3364895; doi:10.1186/1741-7007-10-28)
Supplement: Additional file 1 — FluBO nucleotide and amino acid sequence. Yellow, YFP sequence; black, linker sequence; green, FbFP sequence; grey, His6 tag sequence. [file 1741-7007-10-28-S1.DOC]

**Additional file 1**

FluBO

5’-

CATATGGTGAGCAAGGGCGAGGAGCTGTTCACCGGGGTGGTGCCCATCCTGGTCGAGCTG

M V S K G E E L F T G V V P I L V E L

<*Nde*I>------------------------------------------------------

GACGGCGACGTAAACGGCCACAAGTTCAGCGTGTCCGGCGAGGGCGAGGGCGATGCCACC

D G D V N G H K F S V S G E G E G D A T

------------------------------------------------------------

TACGGCAAGCTGACCCTGAAGTTCATCTGCACCACCGGCAAGCTGCCCGTGCCCTGGCCC

Y G K L T L K F I C T T G K L P V P W P

------------------------------------------------------------

ACCCTCGTGACCACCTTCGGCTACGGCCTGCAGTGCTTCGCCCGCTACCCCGACCACATG

T L V T T F G Y G L Q C F A R Y P D H M

------------------------------------------------------------

AAGCAGCACGACTTCTTCAAGTCCGCCATGCCCGAAGGCTACGTCCAGGAGCGCACCATC

K Q H D F F K S A M P E G Y V Q E R T I

------------------------------------------------------------

TTCTTCAAGGACGACGGCAACTACAAGACCCGCGCCGAGGTGAAGTTCGAGGGCGACACC

F F K D D G N Y K T R A E V K F E G D T

-------------------------YFP--------------------------------

CTGGTGAACCGCATCGAGCTGAAGGGCATCGACTTCAAGGAGGACGGCAACATCCTGGGG

L V N R I E L K G I D F K E D G N I L G

------------------------------------------------------------

CACAAGCTGGAGTACAACTACAACAGCCACAACGTCTATATCATGGCCGACAAGCAGAAG

H K L E Y N Y N S H N V Y I M A D K Q K

------------------------------------------------------------

AACGGCATCAAGGTGAACTTCAAGATCCGCCACAACATCGAGGACGGCAGCGTGCAGCTC

N G I K V N F K I R H N I E D G S V Q L

------------------------------------------------------------

GCCGACCACTACCAGCAGAACACCCCCATCGGCGACGGCCCCGTGCTGCTGCCCGACAAC

A D H Y Q Q N T P I G D G P V L L P D N

------------------------------------------------------------

CACTACCTGAGCTACCAGTCCGCCCTGAGCAAAGACCCCAACGAGAAGCGCGATCACATG

H Y L S Y Q S A L S K D P N E K R D H M

------------------------------------------------------------

GTCCTGCTGGAGTTCGTGACCGCCGCCGGGATCACTCTCGGCATGGACGAGCTGTACAAG

V L L E F V T A A G I T L G M D E L Y K

-----------------------------------------------------------<

GAGCTCGCGGGCCTGGTGCCGCGCGGCAGCGGCGCCGTCGAC*ATG*GCGTCGTTCCAGTCG

E L A G L V P R G S G A V D M A S F Q S

>-----------------Linker-----------------<>-----------------

TTCGGCATCCCGGGCCAGCTGGAAGTCATCAAGAAGGCGCTGGATCACGTGCGCGTCGGC

F G I P G Q L E V I K K A L D H V R V G

------------------------------------------------------------

GTGGTCATCACCGATCCCGCGCTGGAAGATAACCCGATCGTCTACGTGAACCAGGGCTTC

V V I T D P A L E D N P I V Y V N Q G F

------------------------------------------------------------

GTGCAGATGACCGGCTACGAGACCGAGGAAATCCTGGGCAAGAACGCGCGCTTCCTCCAG

V Q M T G Y E T E E I L G K N A R F L Q

-------------------------FbFP-------------------------------

GGGAAGCACACCGATCCGGCGGAAGTGGACAACATCCGCACCGCGCTGCAAAATAAAGAA

G K H T D P A E V D N I R T A L Q N K E

------------------------------------------------------------

CCGGTCACCGTGCAGATCCAGAACTACAAGAAGGACGGCACGATGTTCTGGAACGAACTG

P V T V Q I Q N Y K K D G T M F W N E L

------------------------------------------------------------

AACATCGATCCGATGGAAATCGAGGATAAGACGTATTTCGTCGGCATCCAGAACGACATC

N I D P M E I E D K T Y F V G I Q N D I

------------------------------------------------------------

ACCAAGCAGAAGGAATATGAAAAGCTGCTCGAGCACCACCACCACCACCAC-3’

T K Q K E Y E K L L E H H H H H H

--------------------------><*Xho*I><---His-Tag------>

Additional file 1
