# Supplementary figures and images for: Real-time determination of intracellular oxygen in bacteria using a genetically encoded FRET-based biosensor
Source: BMC Biol. 2012 Mar 22;10:28. doi: 10.1186/1741-7007-10-28 (PMC3364895; doi:10.1186/1741-7007-10-28)

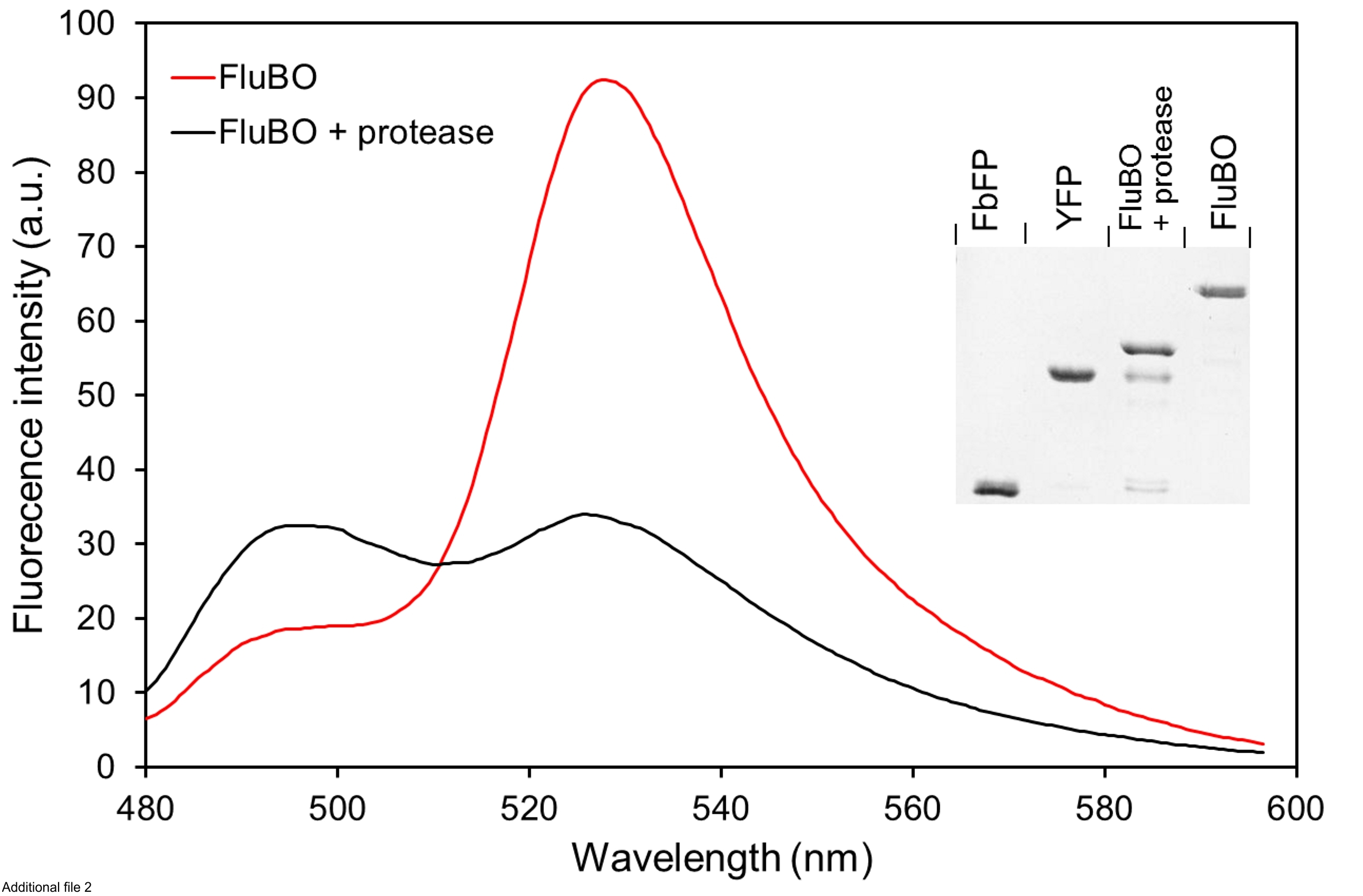

Supplement: Additional file 2 — Fluorescence spectra of FluBO before and after proteolysis with thrombin. Fluorescence spectra of FluBO before and after proteolysis with thrombin. The fluorescence emission spectra of purified FPs (5 μM) were recorded at an excitation wavelength of 380 nm. Complete thrombin-catalyzed proteolysis of FluBO was demonstrated by SDS-PAGE (inset), purified FPs were used as standards. [file 1741-7007-10-28-S2.JPEG]

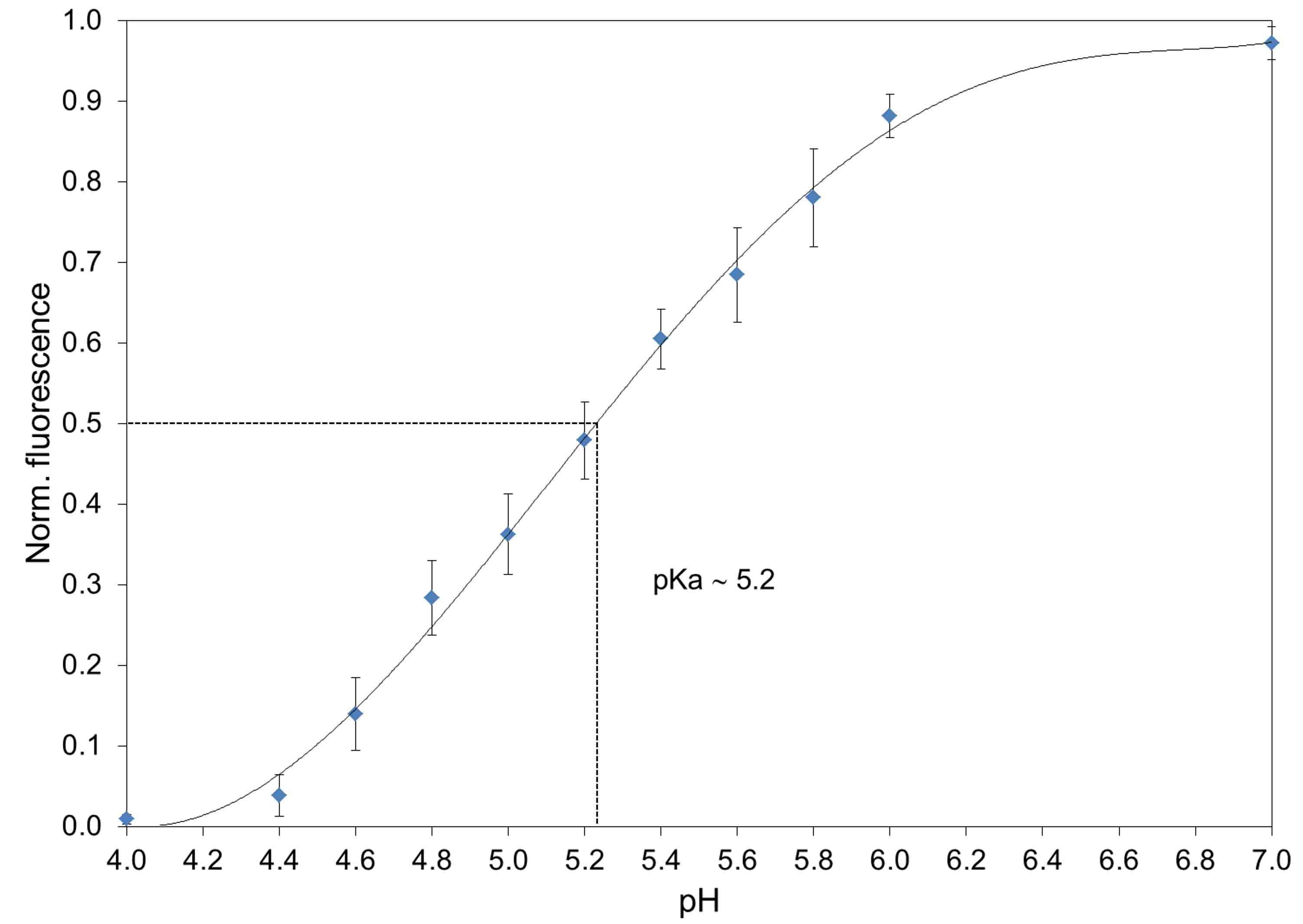

Supplement: Additional file 3 — Sensitivity of used YFP towards pH. The figure shows the normalized YFP fluorescence intensity at increasing pH values. Purified YFP was adjusted to an absorption of 0.05. The effect of the pH value on YFP fluorescence was measured in citric acid/disodium phosphate buffer with pH values ranging form 4.0 to 7.0. The pKa value is marked by doted lines. [file 1741-7007-10-28-S3.JPEG]

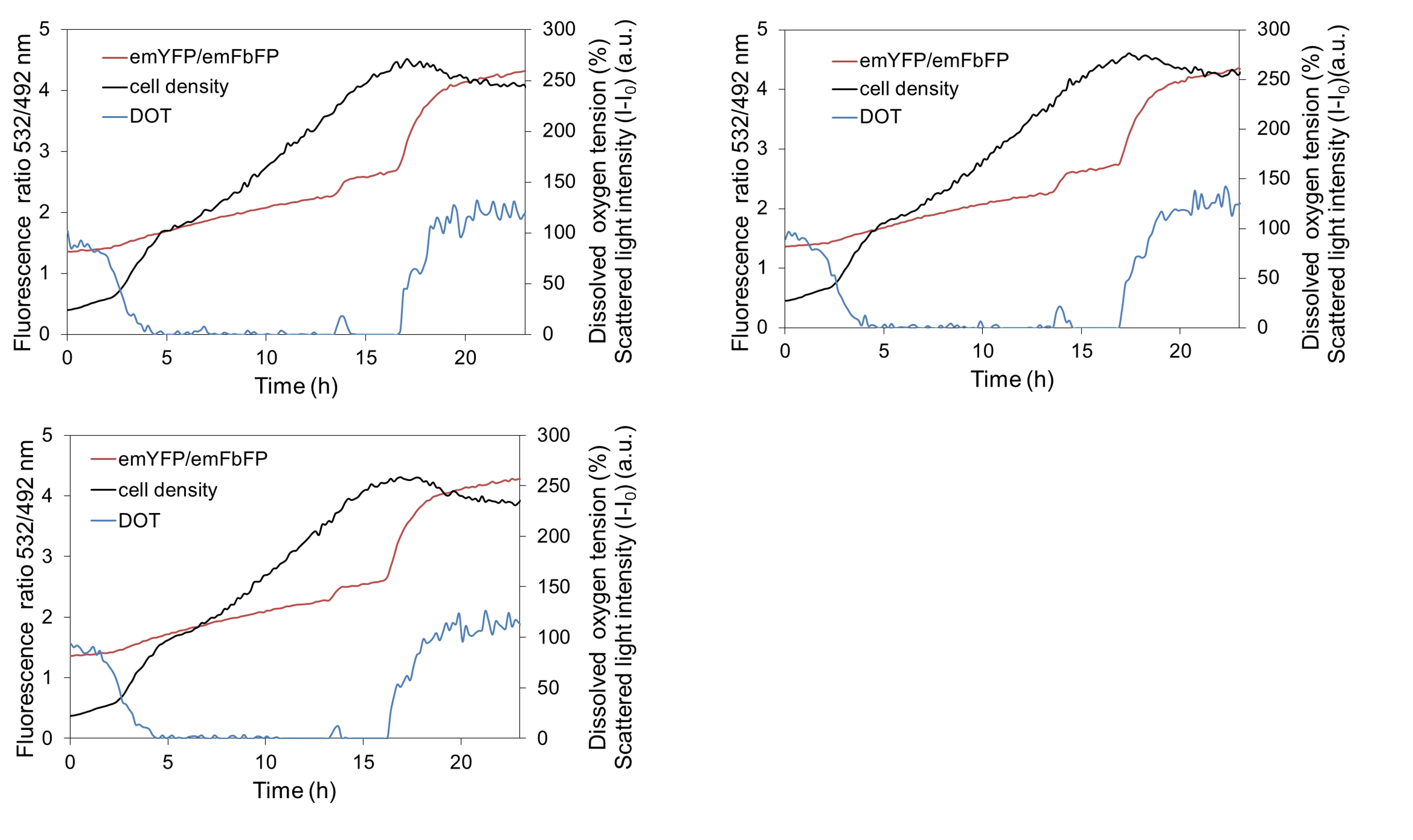

Supplement: Additional file 4 — Data from parallel fermentation experiments. The figure shows the individual results from three independent batch cultivation experiments using the BioLector. The development of biomass (black), dissolved oxygen tension (blue) and cyan-to-yellow fluorescent ratio (emYFP/emFbFP) of the FRET-based oxygen biosensor (red) was online-monitored in each well of the flowerplate. [file 1741-7007-10-28-S4.JPEG]
